# Visually identified breakpoints in muscle oxygen saturation are not equivalent to ventilatory thresholds in national-level triathletes

**DOI:** 10.1007/s00421-026-06357-9

**Published:** 2026-08-17

**Authors:** Ignacio Fernández-Jarillo, Jordi Montraveta, Xavier Iglesias, Chris McManus, Philip Skotzke, Diego Chaverri

**Affiliations:** 1 Programa de doctorat en Activitat Física, Educació Física i Esport, Facultat d’Educació, Universitat de Barcelona (UB), 08035 Barcelona, Spain; 2 Institut Nacional d’Educació Física de Catalunya (INEFC), Universitat de Barcelona (UB), 08038 Barcelona, Spain; 3 School of Sport, Rehabilitation and Exercise Sciences, University of Essex, Colchester, UK; 4 Department of Movement, Human and Health Sciences, University of Rome “Foro Italico”, Rome, Italy

**Keywords:** NIRS, Muscle oxygenation breakpoints, Exercise prescription, Running

## Abstract

**Purpose:**

Portable near-infrared spectroscopy (NIRS) enables cost-effective monitoring of muscle oxygen saturation (SmO_2_) during exercise, presenting a promising alternative for delineating exercise intensity domains. This study investigated whether SmO_2_ breakpoints (BP_1_ and BP_2_) can provide accurate estimates of ventilatory thresholds (GET and RCP).

**Methods:**

Twelve male Tier 3 triathletes completed an outdoor incremental TestVAM. Vastus lateralis SmO_2_ was recorded using NIRS, alongside pulmonary gas exchange measurements. BP_1_ and BP_2_ were identified visually. Heart rate (HR) and running speed at BP_1_, BP_2_, GET, and RCP were extracted. The intraclass-correlation coefficient (ICC) was calculated and equivalence between SmO_2_ breakpoints and ventilatory thresholds was assessed with equivalence bounds set at 5 bpm for HR and 0.5 km h^−1^ for speed.

**Results:**

The ICC(3,1)_abs_ was poor to moderate for all comparisons (range: 0.18–0.69). HR and speed between BP_1_ and GET were not equivalent (*p* > 0.91), with GET occuring ealier in both HR (*p* = 0.014, Δ = − 9.92 bpm, 90% CI [ − 16, − 3.83]) and speed (*p* = 0.007, Δ = − 1.29 km h^−1^, 90% CI [ − 1.99, − 0.59]). Similarly, BP_2_ and RCP were not equivalent (*p* > 0.98), and both HR (Δ = − 9.3 bpm, 90% CI [ − 12.18, − 6.42]) and speed (Δ = − 1.75 km·h^−1^, 90% CI [ − 2.33, − 1.17]) occuring earlier at RCP (*p* < 0.001).

**Conclusion:**

In this sample, SmO_2_ breakpoints did not fullfill criterion validity against ventilatory thresholds and therefore should not be used for determining exercise intensity domains. Further research should evaluate their equivalence using robust detection methodologies in larger samples.

## Introduction

To optimise athletic performance, exercise intensity zones typically are tailored to individual physiological thresholds, which serve as indicators of an athlete's capacity to sustain different intensities of exercise (Wolpern et al. 2015). Among these thresholds, the gas exchange threshold (GET) and the respiratory compensation point (RCP) are well-established. GET is commonly used to operationally define the transition between the moderate and heavy intensity domains (Jamnick et al. 2020). Complementary, RCP is used to demarcate the transition toward the severe intensity domain (Keir et al. 2022). These thresholds provide valuable insights for training prescription and performance monitoring.

Traditionally, GET and RCP are identified using laboratory-based incremental exercise tests requiring gas analysers (Bentley et al. 2007); although portable systems have expanded field testing, their discomfort during exercise and lower accuracy and reliability than most stationary devices still limit widespread adoption (Van Hooren et al. 2024). In this context, near-infrared spectroscopy (NIRS) has emerged as a promising non-invasive, relatively cost-effective alternative for real-time monitoring of muscle oxygen saturation (SmO_2_) during exercise (Belardinelli et al. 1995). SmO_2_ reflects the balance between muscular oxygen (O_2_) availability and consumption and, compared with other NIRS-derived indices used to infer O_2_ extraction (e.g., deoxygenated haemoglobin and myoglobin; deoxy[heme]) (Boone et al. 2016), is considered less influenced by changes in blood volume (Kime et al. 2013; Quaresima and Ferrari 2009).

Early NIRS studies of incremental exercise linked the onset of muscle deoxygenation with both GET and the first lactate threshold (LT_1_) (Belardinelli et al. 1995; Grassi et al. 1999), and Bhambhani et al. (1997) described a triphasic NIRS response that was later confirmed by Spencer et al. (2012). In Phase A, SmO_2_ remains stable despite increasing workload or VO_2_, consistent with an adequate (or surplus) matching of local O_2_ delivery to demand. In Phase B, SmO_2_ declines linearly as workload increases, indicating greater reliance on O_2_ extraction and potentially relative reduced dependence on convective O_2_ delivery. In Phase C, SmO_2_ often plateaus even as pulmonary VO_2_ and external power output continue to rise; this does not necessarily indicate a limit to muscular O_2_ extraction given evidence of an O_2_ extraction reserve (Inglis et al. 2017), but likely reflects interacting mechanisms, including redistribution of blood flow toward the most active tissues, enhancing local O_2_ delivery (Murias et al. 2013) and metabolite-driven vasodilation that further improves perfusion (Inglis et al. 2017). Collectively, this response unfolds in three phases separated by two physiological breakpoints (BP_1_ between Phases A and B, and BP_2_ between Phases B and C).

Muscle oxygenation breakpoints relate to ventilatory thresholds through well-established mechanisms. At GET, an elevated glycolytic flux increases hydrogen ion (H⁺) concentration, reducing bicarbonate and generating non-metabolic CO_2_, which drives a compensatory rise in ventilation relative to VO_2_ (Robergs et al. 2004; Rossiter 2011). Concurrently, acidosis shifts the O_2_ dissociation curve rightward (Bohr effect), facilitating O_2_ offloading (Stringer et al. 1994). These mechanisms may explain why the onset of sustained muscle deoxygenation observed with NIRS (BP_1_) has been associated with GET (Feldmann et al. 2022; Legrand et al. 2007; Belardinelli et al. 1995). As intensity increases beyond GET toward RCP, glycolytic reliance further increases (Bertuzzi et al. 2013), amplifying H⁺ and CO_2_ production and eliciting a stronger ventilatory response (hyperventilation) associated with the second ventilatory threshold (Poole et al. 2021; Galán-Rioja et al. 2020). NIRS similarly detects a shift linked to this transition, termed BP_2_ (Feldmann et al. 2022; Yogev et al. 2022; Legrand et al. 2007; Spencer et al. 2012). Despite the growing body of research, inconsistencies persist in the SmO_2_ breakpoint literature. Several studies locate BP_2_ within Phase B rather than at the Phase B–Phase C transition, effectively splitting Phase B into two segments with a steeper late-phase desaturation slope. Miura et al. (1998) attributed this to myoglobin desaturation, but this explanation is questionable as haemoglobin and myoglobin deoxygenate on a similar time course (Davis and Barstow 2013). Because a steeper Phase B slope is thought to reflect greater O_2_ extraction and/or reduced convective O_2_ delivery (Spencer et al. 2012), further steepening would imply even stronger reliance on these mechanisms; thus, BP_2_ should correspond to the Phase B–Phase C turning point, where the SmO_2_ trajectory shifts from a linear decline to a levelling-off, rather than an intra–Phase B segmentation.

This misplacement is often driven by breakpoint-detection approaches based on curve fitting (e.g., maximal distance, segmented linear regression) (Sendra-Pérez et al. 2023), which optimise statistical fit and can therefore select a slope change within Phase B rather than the physiological Phase B–Phase C transition. Consistent with this, the choice of analytical method can substantially influence outcomes, with reported correlations and standard errors of estimate between blood lactate and SmO_2_-derived threshold power output varying across approaches (Cayot et al. 2021). Moreover, goodness-of-fit indices describe model fit but not physiological validity (Spencer et al. 2012). Accordingly, breakpoint identification should be guided by physiologically meaningful phase transitions and allows for cases in which BP_1_ and/or BP_2_ is absent due to individual differences, muscle activation, or protocol characteristics. Notably, Sendra-Pérez et al. (2023) suggested that visually informed identification, supported by an understanding of the underlying phases, may improve SmO_2_ breakpoint identification. A second issue relates to nomenclature. Among studies using SmO_2_ with sensors placed on the vastus lateralis (VL), some identified two breakpoints (Feldmann et al. 2022; Legrand et al. 2007), while others identified only one (Yogev et al. 2022; van der Zwaard et al. 2016; Rodrigo-Carranza et al. 2021; Raleigh et al. 2018) without a clear physiological justification behind the decision to do so.

Correlational studies have found moderate to strong associations between NIRS breakpoints (BPs) and traditional ventilatory thresholds (VTs) such as GET/VT_1_ and RCP/VT_2_ (Belardinelli et al. 1995; van der Zwaard et al. 2016; Rodrigo-Carranza et al. 2021; Yogev et al. 2022). The use of correlation coefficients has several limitations. They assess association and therefore provide evidence of concurrent validity: that is, whether individuals with higher ventilatory thresholds also tend to show higher NIRS-derived breakpoints (Currell and Jeukendrup 2008). Alternative coefficients, such as the intraclass correlation coefficient (ICC) or Lin’s concordance correlation coefficient, are frequently used to account for error sources not captured by Pearson’s product-moment correlation (Koo and Li 2016; Lin 1989; Weir 2005). However, these coefficients only represent the ratio of explained versus total variance and are affected by sample heterogeneity and size (Nevill and Atkinson 1997; Koo and Li 2016; Weir 2005). Thus, although consistency and low explained variance between methods are a prerequisite for criterion validity, these statistics do not establish whether systematic differences are small enough for one method to substitute the other (DeVellis and Thorpe 2021). To quantify the absolute agreement between NIRS breakpoints and whole-body thresholds, Bland–Altman plots and the Limits of Agreement method commonly are used. This analysis has revealed low mean bias but wide limits of agreement between NIRS BPs and the reference threshold, reflecting limited precision of individual-level estimation (Yogev et al. 2022; Rodrigo-Carranza et al. 2021; Raleigh et al. 2018). These investigations provide a foundation for advancing research on the use of NIRS breakpoints as proxies for estimating whole-body physiological thresholds; however, the key unresolved question is whether SmO_2_ breakpoints can validly estimate criterion thresholds (e.g., GET/RCP) and, by extension, support intensity-prescription frameworks anchored to those thresholds. Addressing this requires more than descriptive agreement, because limits of agreement quantify differences versus a “gold standard” but do not formally test criterion validity. A practical alternative is equivalence testing, which evaluates whether methods yield statistically equivalent results within a-priori acceptable bounds (Lakens et al. 2018). Eserhaut et al. (2025) first applied equivalence testing and reported that SmO_2_ breakpoints derived from piecewise linear regression were not statistically equivalent to blood lactate thresholds in rowers; to our knowledge, no studies have yet applied equivalence testing to directly compare SmO_2_ breakpoints with ventilatory thresholds.

Therefore, the purpose of this exploratory study was to evaluate whether SmO_2_ breakpoints (BP_1_ and BP_2_) meet the requirements for criterion validity against ventilatory thresholds. We compared BP_1_ and BP_2_ with ventilatory thresholds (GET and RCP) identified during a continuous incremental running test (TestVAM) on a 400 m athletics track. We hypothesized that SmO_2_ breakpoints (BP_1_ and BP_2_) and ventilatory thresholds (GET and RCP) would be equivalent, supporting interchangeable use. Within the intersection–union framework, this hypothesis would be accepted only if both speed and heart rate (HR) at the thresholds are statistically equivalent.

## Materials and methods

### Participants

Twelve male Tier 3 triathletes (McKay et al. 2022) participated in the study (Table 1). Inclusion criteria required participants to be licensed and actively competing in national-level triathlon events. Exclusion criteria included any current or recent musculoskeletal injury (within the past two months), known cardiac conditions, or adipose tissue thickness (ATT) > 7 mm, as excessive ATT can attenuate near-infrared light and compromise SmO_2_ measurement accuracy (McManus et al. 2018). ATT was calculated as 0.5 × mean skinfold thickness (Barstow 2019). All participants signed an informed consent form, and the study was approved by the Clinical Research Ethics Committee of the Catalan Sports Administration (026/CEICGC/2023).

**Table 1:** Participant characteristics

Characteristic	N = 12
Age (years)	23.8 ± 5.1 [19.0–32.0]
Height (cm)	175.9 ± 8.5 [167.0–196.0]
Weight (kg)	67.4 ± 6.4 [56.0–78.0]
ATT LVL (mm)	3.8 ± 1.4 [1.6–7.0]
ATT RVL (mm)	3.6 ± 1.5 [1.5–7.0]
TestVAM duration (min)	23.3 ± 1.7 [21.0–26.0]
Vpeak (km h^−1^)	18.8 ± 0.8 [17.5–20.0]
MAS (km h^−1^)	17.8 ± 1.0 [16.0–19.5]
VO_2_max (ml kg^−1^·min^−1^)	62.1 ± 9.3 [47.8–75.0]
BMI	21.8 ± 1.3 [19.4–23.4]

Data are presented as mean ± SD and range. BMI (body mass index), ATT RVL (adipose tissue thickness of the right vastus lateralis) and ATT LVL (adipose tissue thickness of the left vastus lateralis), Vpeak (peak velocity), MAS (maximal aerobic speed), VO_2_max (maximal oxygen uptake)

### Experimental design

Participants completed a single experimental session on an athletics track in Barcelona in environmental conditions of 21.1 ± 5.7 °C and 74.6 ± 16.6% humidity. In the 24 h before testing, they refrained from high-intensity or high-volume sessions. Athletes followed their habitual pre-competition carbohydrate intake and consumed their last meal three hours prior to testing. The session included one exercise task to collect ventilatory, SmO_2_ and HR data. Participants wore a loose running shirt and non-compressive shorts. A portable gas analyser was secured on their back with the mask size selected to fit the individuals face. Participants completed the TestVAM protocol. No warm-up was performed due to the test’s low initial workload. The run took place anticlockwise on a 400 m track, with speed paced by an acoustic signal. Starting at 8 km h^−1^ for two minutes, the speed increased by 0.5 km h^−1^ per minute (Benhammou et al. 2021). Cones were placed every 20 m for pacing, with researchers positioned around the track for monitoring. The test ended when participants voluntarily stopped or failed to reach the marked cone twice in succession.

### Procedures

Body mass and stature were self-reported via questionnaire. Skinfold thickness was measured at the VL using Harpenden callipers (Baty Int., UK). HR was recorded using a Polar H10 monitor (Polar Electro, Kempele, Finland). Muscle oxygenation was monitored using the Moxy Monitor (Fortiori Design LLC, Fort Collins, CO, USA), which measures SmO_2_ on an *a-priori* 0%–100% scale (Feldmann et al. 2019). The Moxy system employs four infrared wavelengths (680, 720, 760, 800 nm) with a single LED and two detectors at 12.5 mm and 25.0 mm from the light source. Sensors were placed on the right and left VL, midway between the greater trochanter and lateral epicondyle (McManus et al. 2024). The measurement site was shaved, and participants were asked not to apply any skin products on the day of testing. The Moxy device was affixed to the skin using waterproof adhesive tape and enclosed with the Moxy Light Shield, a flexible polyurethane covering designed to block external light sources that could disrupt the signal. The device operated in its default configuration, cycling through four wavelengths 80 times every 2 s, resulting in an output sampling rate of 0.5 Hz. Participants wore a dead-space mask (Hans Rudolph, USA) with a 28 mm bidirectional digital turbine. O_2_ and CO_2_ concentrations were measured breath-by-breath (Cosmed S.r.l, Albano Laziale, Rome, Italy). The gas analyser was calibrated following the manufacturer guidelines, including room air, flow meter (3-L syringe), scrubber, reference gas (16% O_2_, 5% CO_2_), and delay calibrations.

### Data analysis

Data were collected using Cosmed Omnia (version 2.3) and Moxy Settings App (version 1.5.5) and processed in Microsoft Excel (version 16.81 24011420). Both devices were time-synched using the starting time of recordings. To account for the circulatory transit delay between muscle and lungs, ventilatory data were left-shifted by 20 s for time-alignment with SmO_2,_ consistent with previous investigations (Murias et al. 2013). Ventilatory, HR and SmO_2_ data were averaged over 10-s intervals, using non-overlapping 10-s means (one value every 10 s). The ten-second bins were defined as 1–10, 11–20, 21–30 s, etc., and values were right-aligned to the end of each bin.

Peak VO_2_ was determined as the highest 30-s average VO_2_ value during the test. Vpeak was defined as the highest speed attained, while maximal aerobic speed (MAS) was identified as the lowest speed that elicited VO_2_max (Pallarés et al. 2019). GET was identified using the V-slope method, based on a disproportionate increase in CO_2_ excretion rate (VCO_2_) relative to VO_2_. It was confirmed by an increase in the ventilatory equivalent of O_2_ uptake (VE/VO_2_) and the end-tidal pressure of O_2_ (PETO_2_) without a concurrent rise in the ventilatory equivalent of VCO_2_ (VE/VCO_2_) or the end-tidal pressure of CO_2_ (PETCO_2_). RCP was determined at the point of inflection in VE/VCO_2_ and the second inflection in VE/VO_2_. Confirmation was based on a deviation from stability in VE/VCO_2_, a steeper rise in VE/VO_2_, and a decrease in PETCO_2_ (Keir et al. 2022). Both GET and RCP were identified independently by two experienced observers with disagreement settled by consensus. Once determined, HR at GET/RCP was extracted from the corresponding 10-s HR bin. Velocity at GET/RCP was taken as the stage velocity; when GET or RCP occurred at a stage boundary (60 s or multiples of 60 s), velocity was assigned to the preceding stage to match the right-aligned bins.

**Fig. 1:**
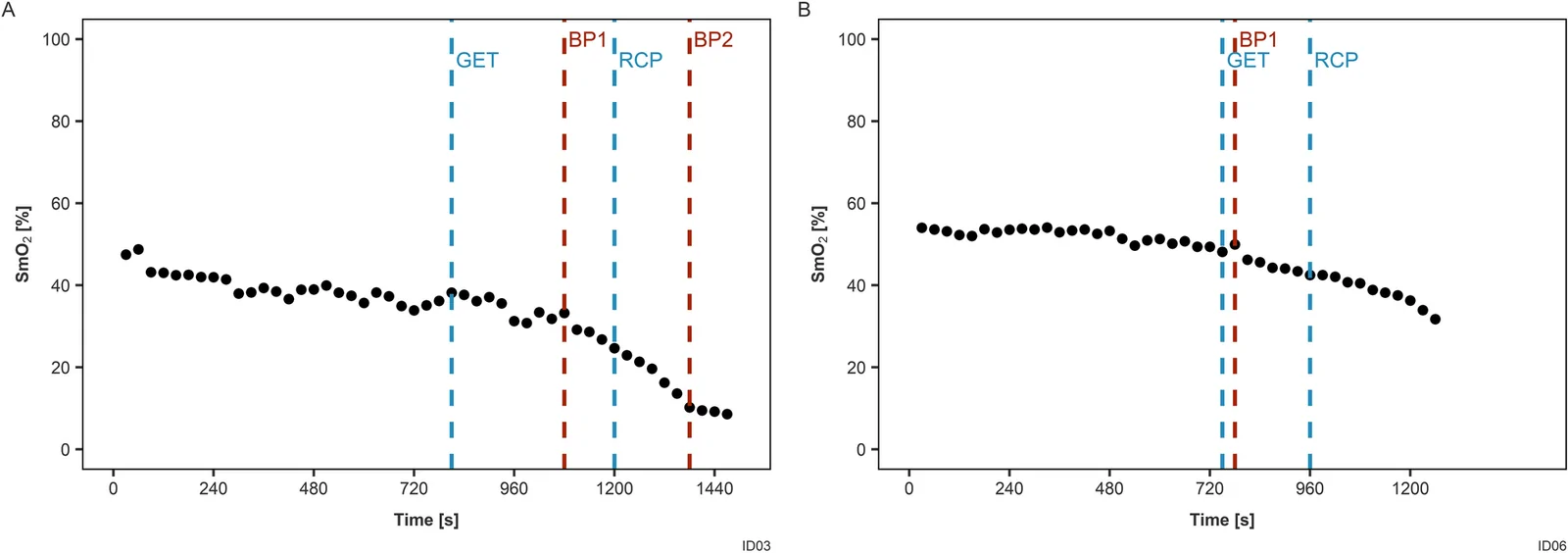
Breakpoint detection in one representative participant with both BP_1_ and BP_2_ (left panel) and one participant with BP_1_ only (right panel). Note. Black dots indicate SmO_2_ (%) data points recorded throughout the TestVAM, while red dotted lines denote the identified SmO_2_ breakpoints (BP_1_ and BP_2_) and blue dotted lines denote the identified ventilatory thresholds (GET and RCP) for this individual. For this figure, the y-axis was scaled to 0–100%, making breakpoints appear mor subtle

For SmO_2_ breakpoint identification, SmO_2_ data were averaged over 30-s intervals resulting in two data points per stage of the TestVAM for each variable. SmO_2_ 30-s values were obtained by averaging three consecutive 10-s bins and were right-aligned to the end of each 30-s interval. For the analysis, values from the right VL were reported. When a breakpoint could not be identified in the right VL, left VL data were used. All BP_1_ were detected in the right VL. For BP_2_, 9 of 12 were identified in the right VL, and among the three subjects in whom BP_2_ was not detected in the right VL, it was possible to identify BP_2_ in the left VL in one case. Breakpoints in SmO_2_ were identified through visual inspection by two experienced observers with disagreement settled by consensus. SmO_2_ was plotted against time, and the y-axis scaled to optimally cover the SmO_2_ range of each test, allowing precise identification of the breakpoints. BP_1_ was defined as the last data point before SmO_2_ entered a sustained decline i.e., the final point where no further increases in SmO_2_ were observed (Rodrigo-Carranza et al. 2021). BP_2_ was defined as the data point immediately preceding a positive inflection in the deoxygenation response that characterized the post-BP_1_ phase (Legrand et al. 2007) (Fig. 1). Following identification of BP_1_ and BP_2_, HR was paired using a 30-s mean aligned to the same 30-s interval as SmO_2_. Because speed increased every 60 s, values at 60 s (or multiples of 60 s) were assigned the speed of the preceding stage.

### Statistical analysis

The statistical analysis was performed using R (version 4.5.2) in RStudio (version 2025.09.1.401). First, to investigate the reliability of breakpoints versus ventilatory thresholds, the ICC(3,1) for absolute agreement was calculated (Koo and Li 2016) using the {SimplyAgree} package (version 0.3.0). Prior to equivalence testing, the paired differences were screened for assumption violations: Bland–Altman style plots were inspected for heteroscedasticity and Q-Q plots and the Shapiro–Wilk test were used to assess normality. Limits of agreement were not derived, as the inferential assessment of equivalence was based on the equivalence test against pre-specified, clinically anchored bounds rather than on descriptive agreement intervals. Two one-sided tests (TOST) were used to test statistical equivalence between BP_1_ and GET, and between BP_2_ and RCP using the {TOSTER} package (version 0.8.6). Paired TOSTs were performed together with a null hypothesis significance test. NIRS breakpoints and ventilatory thresholds were considered equivalent if both the TOST for HR and the TOST for speed are significant. Thus, within this intersection–union approach, no α correction is needed and the alternative hypothesis is accepted only if both the TOST for HR and speed are significant (Mesquida et al. 2025). As equivalence bounds and smallest effect size of interest, 5 bpm for HR and 0.5 km h^−1^ for speed were chosen. These provide an empirically grounded justification (Lakens et al. 2018) based on the test–retest reliability of the ventilatory thresholds of 4–5 bpm for HR (Weston and Gabbett 2001) and 0.2–0.4 km h^−1^ for speed (Cerezuela-Espejo et al. 2018). The acceptable Type I error rate was specified a priori at α = 0.05. The data, statistical code and results are openly available on OSF: (https://osf.io/u8k2e/).

## Results

**Fig. 2:**
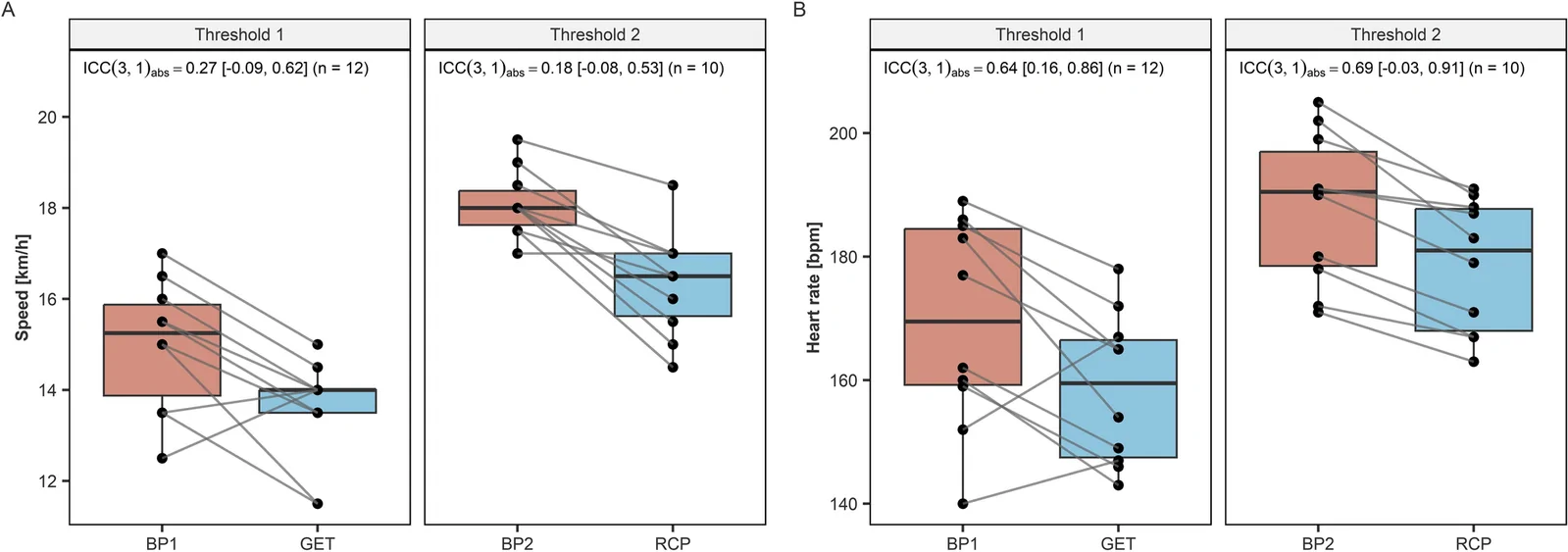
Group level and individual athlete data for the **A** speed and **B** heart rate at SmO_2_ breakpoints and ventilatory thresholds. Intraclass correlation coefficients [ICC(3,1), absolute agreement] with 95% confidence intervals are displayed for each comparison

Visual inspection of the Bland–Altman plots revealed no substantial heteroscedasticity in the differences between NIRS-derived breakpoints and ventilatory thresholds for either heart rate or speed. The ICCs (version 3,1, absolute agreement) for each comparison are presented alongside the corresponding breakpoints and ventilatory thresholds in Fig. 2. For heart rate at BP_1_ and GET the ICC was 0.64 (95% CI [0.16,0.86]), indicating poor to moderate agreement. For the remaining comparisons, speed at BP_1_ and GET (ICC = 0.27, 95% CI [ − 0.09, 0.62]), heart rate at BP_2_ versus RCP (ICC = 0.69, 95% CI [ − 0.03, 0.91]), and speed at BP_2_ versus RCP (ICC = 0.18, 95% CI [ − 0.08, 0.53]), the point estimates ranged from poor to moderate, and all 95% confidence intervals extended below zero. Across all four comparisons the confidence intervals were wide, indicating that the present sample did not show reliable agreement between NIRS-derived breakpoints and ventilatory thresholds.

### Equivalence

#### BP_1_ and GET

**Fig. 3:**
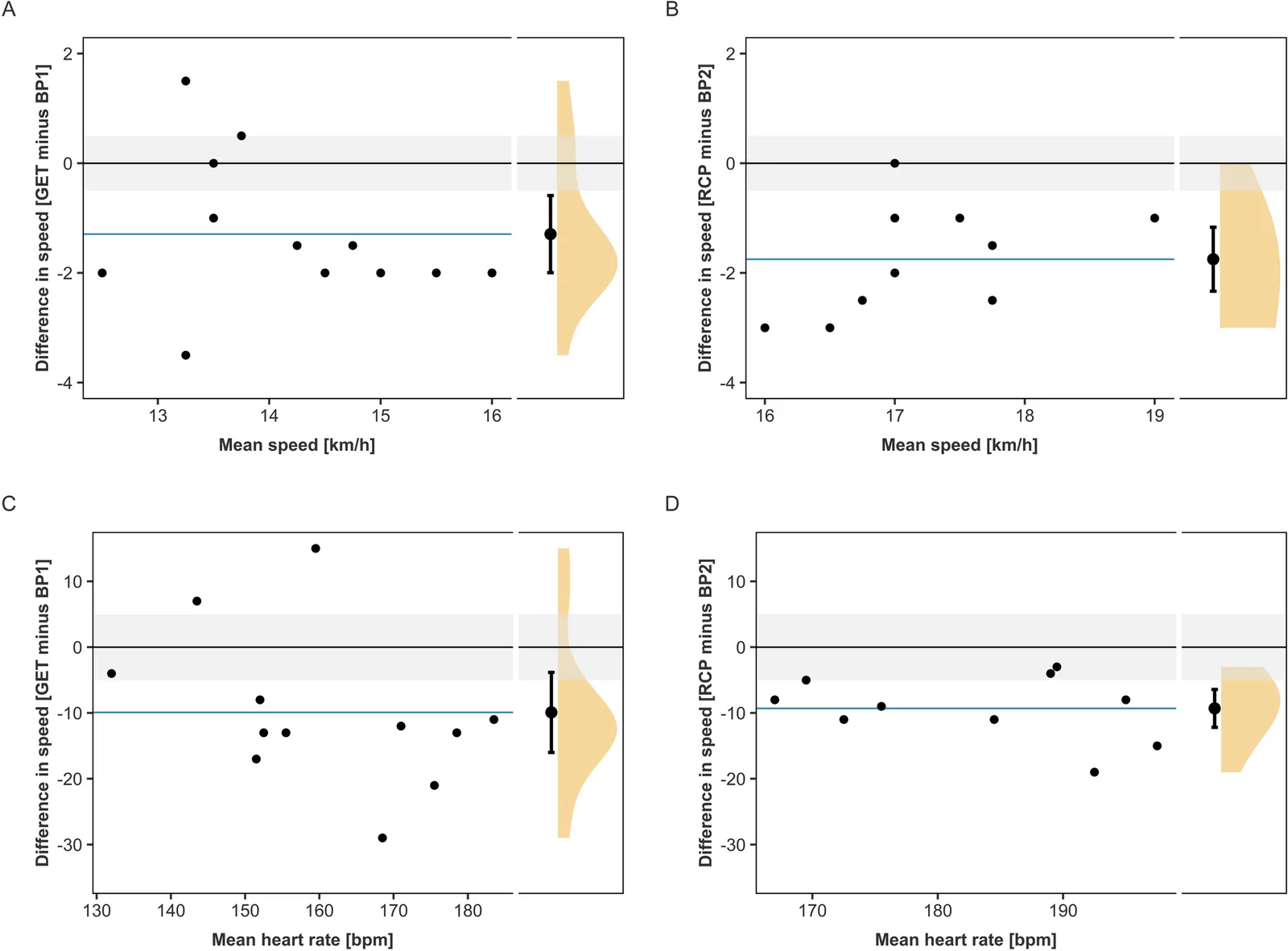
Bland–Altman style plots (left part of each panel) and equivalence plots (right part of each panel) for **A** speed at BP_1_ versus GET, **B** speed at BP_2_ versus RCP, **C** heart rate at BP_1_ versus GET, and **D** heart rate at BP_2_ versus RCP. In the Bland–Altman plots, individual differences are plotted against the mean of the two methods; the blue solid line indicates the mean difference. The equivalence plots show the mean difference with its 90% confidence interval (error bars) and the distribution of differences (density curve). Differences were calculated as ventilatory threshold minus NIRS breakpoint. Grey shaded areas indicate the pre-specified equivalence range

The heart rate and speed at BP_1_, BP_2_, GET and RCP for each athlete are presented in Fig. 2. For HR at the first threshold, the equivalence test was not significant (*p* = 0.913); that is, equivalence within the pre-specified range could not be demonstrated. The corresponding null hypothesis significance test (NHST) was significant, t(11) = -2.93, *p* = 0.014, with a mean difference in heart rate of − 9.92 bpm (90% CI [ − 16.00, − 3.83]). Together, these results indicate a meaningful difference in HR between BP_1_ and GET rather than equivalence between methods. A comparable pattern was found for speed: the equivalence test was not significant (*p* = 0.966) and the NHST was significant, t(11) = − 3.30, *p* = 0.007, with a mean difference of − 1.29 km h^−1^ (90% CI [ − 1.99, − 0.59]). Across both variables GET occurred at a lower speed and heart rate than BP_1_. The results are visualized in Fig. 3.

#### BP_2_ and RCP

For the second physiological threshold, the equivalence test was neither significant for HR (*p* = 0.989) nor speed (*p* = 0.998), indicating that equivalence within the pre-specified margin could not be demonstrated for either variable. In both cases the NHST was significant: t(9) = − 5.92, *p* < 0.001 for HR and t(9) = − 5.50, *p* < 0.001 for speed. The mean difference was − 9.3 bpm (90% CI [ − 12.18, − 6.42]) for HR and − 1.75 km h^−1^ (90% CI [ − 2.33, − 1.17]) in speed. As at the first threshold demarcation, RCP occurred at a lower HR and speed than BP_2_. The results are visualized in Fig. 3.

## Discussion

The present study aimed to test criterion validity of the muscle oxygenation breakpoints against established ventilatory thresholds during an incremental running test performed on an outdoor track. We hypothesized that BP_1_ would be equivalent to GET, and BP_2_ to RCP, in both HR and running speed. Our results indicate that equivalence was not achieved and that the muscle oxygenation breakpoints obtained from the VL were significantly different from the ventilatory thresholds when using the TestVAM protocol and the visual identification method, demonstrating a lack of criterion validity under the present conditions.

Any alternative method proposed as a surrogate for ventilatory thresholds should demonstrate criterion validity against the established reference method. This underscores the value of equivalence testing when evaluating validity of alternative methods for estimating physiological thresholds. The results of the equivalence test do not support the interchangeable use of the different thresholds. In none of the comparisons (BP_1_ vs. GET; BP_2_ vs. RCP) did HR or running speed meet the pre-defined criteria for equivalence. The 90% confidence intervals of the differences did not fall within the pre-defined equivalence bounds (Fig. 3). At the same time, all NHST results were statistically significant, indicating meaningful differences between the thresholds. In line with this systematic difference, the ICC(3,1)_abs_ indicated only low-to-moderate and imprecise agreement between methods. This is expected as the absolute-agreement ICC incorporates the between-methods variance term, so that a systematic difference between methods—as in our case—inflates the denominator and lowers the coefficient. The wide confidence intervals of the ICC estimates, however, indicate that agreement could not be estimated precisely in the present sample, and the ICC point estimates should therefore be interpreted with caution (see Limitations).

Previous studies have proposed that muscle oxygenation breakpoints identified through NIRS may serve as proxies for ventilatory thresholds, suggesting that these breakpoints can be used to infer them, based on agreement analyses or the absence of statistically significant differences (Feldmann et al. 2022; Rodrigo-Carranza et al. 2021). However, these approaches do not provide formal evidence of breakpoint validity. Within a frequentist framework, a non-significant *p*-value is not evidence of equivalence; it indicates insufficient evidence for a difference, not confirmation of practical equivalence. In contrast, equivalence testing in the present study provides a direct assessment of whether the observed differences fall within an acceptable equivalence range. Here, we defined our equivalence range based on the test–retest reliability of ventilatory thresholds, considering differences between methods small enough when they were smaller or equal to the day-to-day variability of the ventilatory thresholds.

Taken together, our findings challenge the view that NIRS-derived breakpoints can serve as direct substitutes for ventilatory thresholds, aligning with evidence that lactate thresholds (LT_1_ and LT_2_) are not equivalent to SmO_2_ breakpoints in rowing (Eserhaut et al. 2025). Furthermore, other studies have raised concerns regarding the interchangeability of NIRS breakpoints and whole-body thresholds (Caen et al. 2018, 2022; Goulding et al. 2021; Reinpõld et al. 2024; Arnet et al. 2025; Tilp et al. 2025). In contrast, a systematic review with meta-analyses concluded that the second breakpoint shows good reliability (Sendra-Pérez et al. 2023), based on a mean weighted intraclass correlation coefficient of 0.80 [ICC(2,1)_abs_] with a 95% confidence interval ranging from 0.65 to 0.89. Because the ICC is a ratio, a high value can coexist with a systematic between-method difference large enough to alter an exercise prescription: in a heterogeneous sample, large between-athlete variance keeps the coefficient high even in the presence of an absolute systematic difference. As outlined in the introduction, the ICC does not test whether differences between methods fall within a clinically acceptable range and thus cannot, on its own, answer the question of interchangeability or criterion validity.

A major factor influencing NIRS breakpoints, and therefore their equivalence with ventilatory thresholds, is the exercise protocol and the breakpoint identification method. Whereas ventilatory threshold detection is well established (Keir et al. 2022), approaches for identifying NIRS breakpoints vary substantially (Sendra-Pérez et al. 2023), and the selected method can affect the outcome (Cayot et al. 2021). Visual identification has been used in several studies (Rodrigo-Carranza et al. 2021; Salas-Montoro et al. 2022) and may be the most accurate and physiologically informed approach (Sendra-Pérez et al. 2023), although the differences observed here may still reflect methodological limitations of visual detection. However, visually identified breakpoints are based on proposed physiological mechanisms, making them interpretable. In contrast, mathematical derived breakpoints typically lack physiological justification. Additional methodological factors may contribute: the vastus lateralis may not be optimal for breakpoint detection in running protocols, as it has been reported to be among the least activated and least intensely recruited muscles during level running (Sloniger et al. 1997). Finally, the TestVAM design consisting of small speed increments (0.5 km h⁻^1^) and short 1-min stages may influence agreement with ventilatory thresholds; smaller increments have been proposed to strengthen GET–NIRS associations (Van der Zwaard et al. 2016), while the role of stage duration on NIRS breakpoint identification and its alignment with ventilatory thresholds remains unclear.

These methodological considerations assume a physiological link between NIRS-derived breakpoints and whole-body thresholds (Boone et al. 2016; Caen et al. 2022; Caen & Boone 2023). If such a link exists, methodological refinements such as improved breakpoint detection, optimized protocol design, or more appropriate muscle selection may enhance the agreement between these measures. However, an alternative explanation for the systematic differences between NIRS breakpoints and ventilatory thresholds is that the physiological mechanisms leading to sudden changes in local SmO_2_ are different from those explaining ventilatory thresholds (Goulding et al. 2023). While our findings do not rule out a mechanistic link between SmO_2_ breakpoints and ventilatory thresholds, they indicate that even if such a link exists, the temporal sequence of events may not align across individuals, and these measures cannot be used interchangeably. Following this line of reasoning, Boone et al. (2016) proposed a conceptual framework in which breakpoints in muscle oxygenation and pulmonary VO_2_ occur within a coordinated cascade of physiological responses, although the sequence appears to be individual-dependent (Caen et al. 2022), and the inter-individual variation could also be random (Goulding et al. 2023).

Eserhaut et al. (2025), using the double linear regression approach, reported that BP_1_ significantly underestimated LT_1_ and that, BP_2_ while not significantly different from LT_2_, preceded it and was not equivalent, suggesting that reduced O_2_ availability within skeletal muscle preceded subsequent blood lactate accumulation. Similarly, Reinpõld et al. (2024), found that BP_1_ significantly underestimated VT_1_ in senior cyclists, a pattern not observed in junior cyclists. In the fitter junior cyclists, BP_1_ and VT_1_ did not differ at the group level, suggesting that a later BP_1_ relative to VT_1_ may reflect more effective local matching of oxygen delivery to utilization and a better-functioning peripheral component. Accordingly, greater fitness may delay the emergence of BP_1_, such that initial deoxygenation occurs at a higher relative intensity; this may partly explain why BP_1_ appeared significantly later than GET in our homogeneous group of Tier 3 triathletes. Moreover, the relatively lower involvement of the VL in level running compared with other lower-limb muscles such as the adductors and semitendinosus (Sloniger et al. 1997) may also contribute to BP_1_ occurring after GET. Although the VL contributes to the metabolic response at GET, it is unlikely to be the primary driver of the whole-body response. This interpretation is supported by Yogev et al. (2022), who observed deltoid desaturation patterns (a non-locomotor muscle during cycling) resembling those found in the VL in the present study, and by Chin et al. (2011), who reported a rightward shift in rectus femoris deoxygenation relative to the VL during cycling that was attributed to lower muscle activation. Similarly, Rodrigo-Carranza et al. (2021), in well-trained runners, found no significant differences between a VL breakpoint analogous to BP_1_ and VT_2_, according to the definition used in the present study. This may reflect both lower VL activation during running and a fitness-related rightward shift in the NIRS breakpoint.

Regarding BP_2_, one study comparing BP_2_ and RCP reported wide limits of agreement between VO_2_ at BP_2_ in the VL and VO_2_ at RCP (exceeding 10 ml kg^−1^ min^−1^), together with substantial individual variability in the timing and sequence of NIRS-derived and systemic thresholds (Yogev et al. 2022). Another study found that BP_2_ preceded RCP, although the difference was not statistically significant (Legrand et al. 2007). Direct comparisons with these studies are limited by methodological differences, including exercise modality (cycling vs. running) and fitness level. Moreover, previous studies used different operational definitions of the BPs (Feldmann et al. 2022; Rodrigo-Carranza et al. 2021), further limiting direct comparison.

Finally, even if NIRS-derived breakpoints cannot be used interchangeably with systemic thresholds, defining them in a physiologically meaningful way may still provide complementary insight into localized peripheral responses over time. Accordingly, studies should explicitly report whether BP_1_, BP_2_, or both were identified, because two distinct breakpoints may emerge within a muscle region during incremental exercise but may not be detectable in every muscle or across all sporting disciplines, as shown in our findings and previous work (Yogev et al. 2022; Rodrigo-Carranza et al. 2021).

## Limitations

A limitation of this study is the small and relatively homogeneous sample, which results in poor precision in statistical estimates and restricts the generalizability of the findings to broader athletic populations. Consequently, the observed difference between SmO_2_ breakpoints and ventilatory thresholds may not reflect those in a more diverse cohort of athletes. In particular, the homogeneity of the sample reduces the ICC point estimates, which are therefore likely conservative and should not be read as direct evidence against agreement between methods. This limitation can also be considered a strength, as the findings are highly applicable to triathletes of comparable performance levels, particularly those classified as Tier 3 (McKay et al. 2022). Only males were tested. It remains unexplored how SmO_2_ breakpoints compare with ventilatory thresholds in women. Sex differences in both the respiratory system and peripheral muscle oxidative capacity are well established (Ansdell et al. 2020). This central limitation in females may lead to ventilatory thresholds occurring at a lower relative intensity while breakpoints in muscle oxygen saturation could occur later than in men.

To account for circulatory transit delay and align VO_2_ with SmO_2_ responses, a 20-s time shift was applied to the ventilatory data. While this approximation has been previously implemented in several investigations (Murias et al. 2013), a fixed offset may not fully capture individual variability in circulatory time lag. Additionally, the speed of each threshold was identified as the corresponding stage. In cases where the BP occurred at the end of the stage and the VT occurred at the beginning of the next stage (or vice versa), the true difference in speed could be inflated by up to 0.5 km h⁻^1^, which is the SESOI of this study. While this did not affect the results in our sample, future studies should linearly interpolate the speed of the thresholds to eliminate this potentially inflated difference. This study used a NIRS device with a fixed inter-optode distance of 25 mm, limiting signals to superficial muscle layers, potentially missing contributions from deeper muscle fibers shown to exhibit different oxygenation dynamics during exercise (Okushima et al. 2015). Future studies employing the same methodological approach but with deeper tissue penetration may come to different conclusions.

Finally, this study makes use of additional data collected as part of a larger research project. As such, this is an exploratory study without an a priori sample size calculation for the present research question, and the sample size was too small to make any claims on the use of NIRS breakpoints as an alternative to ventilatory thresholds with certainty. Based on our results we estimated the required sample size to achieve sufficient (80%) power when applying equivalence testing to compare NIRS breakpoints with ventilatory thresholds. If a NIRS breakpoint method has a true mean difference from the criterion method of 2 bpm in HR and 0.2 km·h^−1^ in velocity, at least 128 participants would be needed to severely test equivalence against the criterion (see the supplementary file on OSF for more details).

## Future directions

The simplicity of collecting NIRS data in field-based settings such as this study make it a promising technology for practitioners beyond the laboratory setting. Still, one of the central challenges in the field of NIRS breakpoint analysis is the substantial methodological heterogeneity across studies (Sendra-Pérez et al. 2023), which directly affects both the results obtained and the conclusions drawn. To address this, we recommend future investigations to adopt the standardized framework proposed in this study for evaluating agreement between NIRS-derived breakpoint groups and whole-body physiological thresholds. We encourage future research to examine how variations in step duration, step increment, and overall protocol design influence the agreement between NIRS-derived breakpoints and whole-body thresholds, given their impact on lactate and ventilatory thresholds (Bentley et al. 2007). Future studies should also determine which muscles yield better agreement between NIRS-derived breakpoints and systemic thresholds, and whether this depends on sport discipline and exercise modality: This is particularly relevant given recent cross-country skiing data suggesting closer agreement between LT_2_ and breakpoints derived from the biceps femoris and biceps brachii than from the commonly monitored vastus lateralis (Forot et al. 2026). This could be addressed by using multiple NIRS probes across muscle groups. Establishing a physiologically justified framework for SmO_2_ breakpoints may provide a robust foundation for the development and validation of objective mathematical methods for breakpoint detection. This sets the basis to investigate predicitve validity (Currell & Jeukendrup 2008) to establish domain-specific phyisological responses during constant-workload exercise as has been argued earlier (Skotzke et al. 2025).

## Conclusion

In this study, we visually identificated SmO_2_ breakpoints in the VL during a field-based running exercise test of Tier 3 triathletes. When compared to ventilatory thresholds, SmO_2_ breakpoints were found to occur at a higher speed and heart rate and not to be equivalent with the established criterion method. Therefore, it cannot be recommended to use NIRS-derived breakpoints interchangeably with ventilatory thresholds, suggesting that SmO_2_ breakpoints—at least when identified with the specific methodology and test protocol used in this study—should not be used as direct substitutes for GET/RCP to anchor training-zone prescription when those zones are defined using ventilatory thresholds.

## Data Availability

The data used in the study is openly available on OSF: https://osf.io/u8k2e/

## Abbreviations

ATT: Adipose tissue thickness
BP: Muscle oxygenation breakpoint
BP_1_: Breakpoint 1
BP_2_: Breakpoint 2
CO_2_: Carbon dioxide
GET: Gas exchange threshold
H +: Hydrogen ion
HR: Heart rate
ICC: Intraclass correlation coefficient
LT_1_: First lactate threshold
LT_2_: Second lactate threshold
MAS: Maximal aerobic speed
NIRS: Near-infrared spectroscopy
O_2_: Oxygen
PETCO_2_: End-tidal pressure of CO_2_
PETO_2_: End-tidal pressure of O_2_
RCP: Respiratory compensation point
SmO_2_: Muscle oxygen saturation
TOST: Two one-sided tests
VL: Vastus lateralis
VCO_2_: Carbon dioxide excretion rate
VE/VCO_2_: Ventilatory equivalent of VCO_2_
VE/VO_2_: Ventilatory equivalent of VO_2_
VO_2_: Oxygen uptake
Vpeak: Peak velocity
VT: Ventilatory threshold
VT_1_: First ventilatory threshold
VT_2_: Second ventilatory threshold
